# Green Synthesis, Molecular Docking, and QSAR Study of Coumarin Derivatives as Acaricidal Agents Against *Rhipicephalus* (*Boophilus*) *microplus*

**DOI:** 10.3390/molecules31173100

**Published:** 2026-09-04

**Authors:** Carlos E. Rodriguez, Diego M. Ruiz, Alejandra Rodriguez, Pablo R. Duchowicz, Gustavo P. Romanelli, José J. Martínez, Laura Juliana Triana Triana

**Affiliations:** 1 Facultad de Medicina Veterinaria y Zootecnia, Universidad Pedagógica y Tecnológica de Colombia UPTC, Tunja 150001, Boyacá, Colombia; alejandrarodriguez@jdc.edu.co (A.R.); laurajuliana.triana@uptc.edu.co (L.J.T.T.); 2 CISAV, Cátedra de Química Orgánica, Facultad de Ciencias Agrarias y Forestales, Universidad Nacional de La Plata, La Plata B1904AAN, Argentina; gpr@quimica.unlp.edu.ar (G.P.R.); 3 Facultad de Ciencias Agrarias y Ambientales, Fundación Universitaria Juan de Castellanos, Tunja 150003, Boyacá, Colombia; 4 Instituto de Investigaciones Fisicoquímicas Teóricas y Aplicadas (INIFTA), CONICET, UNLP, La Plata B1900FWA, Argentina; prduchowicz@yahoo.com.ar; 5 Centro de Investigación y Desarrollo en Ciencias Aplicadas “Dr. Jorge J. Ronco” (CINDECA-CCT La Plata-CONICET), Universidad Nacional de La Plata, La Plata B1904AAN, Argentina; 6 Escuela de Ciencias Químicas, Facultad de Ciencias, Universidad Pedagógica y Tecnológica de Colombia UPTC, Avenida Central del Norte, vía Paipa, Tunja 150001, Boyacá, Colombia

**Keywords:** coumarins, acaricidal activity, *Rhipicephalus* (*Boophilus*) *microplus*, green synthesis, Preyssler heteropolyacid, Pechmann reaction, adult immersion test, cox proportional hazard model, molecular docking, triosephosphate isomerase (TIM), QSAR

## Abstract

Coumarins are heterocyclic compounds with diverse biological activities and potential applications as alternative acaricidal agents. In this study, nine coumarin derivatives were synthesized through a solvent-free Pechmann reaction using Preyssler heteropolyacid as a reusable catalyst under green chemistry conditions. The synthesized compounds were evaluated against adult females of *Rhipicephalus* (*Boophilus*) *microplus* using the adult immersion test. Survival analysis was performed using the Cox proportional hazard model, while molecular docking studies were carried out on triosephosphate isomerase (TIM). In addition, post hoc analysis of the structures predicted to be most active by the QSAR model suggested an association between higher predicted activity, the presence of hydroxyl groups, and the polarity of C7 side chains, although the topological descriptor identified by the model does not admit a direct physicochemical interpretation. The results showed that hydroxylated coumarins, particularly those substituted at C5 and C7, exhibited the highest acaricidal activity, with IC_50_ values between 7.24 and 8.78 mg/mL. Docking analysis suggested plausible interactions with residues located at the TIM interface cavity, mainly Lys-112, Asn-65, and Glu-77. The QSAR model indicated that hydroxyl substitution and side-chain polarity contributed positively to activity. This study integrates Preyssler heteropolyacid-catalyzed green synthesis of coumarins with Cox proportional hazard survival modeling, TIM molecular docking, and QSAR analysis within a single acaricidal evaluation against *R.* (*B.*) *microplus*, providing a multi-pronged framework for prioritizing structural modifications in future coumarin-based acaricide development.

## 1. Introduction

Coumarins are an important class of heterocyclic compounds widely recognized for their diverse biological and industrial applications. Compounds containing the coumarin scaffold have shown antibacterial, antiparasitic, antifungal, anticancer, anti-inflammatory, and antiviral activities, including selective inhibition of HIV-1 protease [1,2,3,4,5,6,7,8]. In addition, coumarin derivatives are extensively used in fragrances, fluorescent dyes, food additives, and optical sensors and as precursors for the synthesis of other biologically active compounds such as chromones and furocoumarins [9,10,11,12,13,14].

Natural and synthetic coumarins have also attracted attention because of their insecticidal and antiparasitic properties. Several studies have reported their activity against lepidopteran insects, Aedes aegypti larvae, and nematodes of the family Trichostrongylidae [15,16]. The biological activity of coumarins is strongly influenced by structural modifications in the α-pyrone ring and the substitution pattern of the aromatic moiety [17]. In particular, naturally occurring coumarins and hydroxylated derivatives such as scopoletin have demonstrated acaricidal activity against arthropod pests, including mites, while other coumarin derivatives have shown activity against termites and other insect species [18,19,20].

The cattle tick *Rhipicephalus* (*Boophilus*) *microplus* is one of the most economically important ectoparasites affecting livestock production worldwide [21]. This hematophagous tick is widely distributed in tropical and subtropical regions and causes significant economic losses through weight reduction, skin damage, decreased milk production, and transmission of pathogens responsible for babesiosis, anaplasmosis, ehrlichiosis, and other diseases [22,23,24,25,26]. Current tick control strategies mainly rely on synthetic acaricides such as organophosphates, pyrethroids, formamidines, and macrocyclic lactones [27]. However, the intensive and indiscriminate use of these compounds has resulted in environmental contamination, residues in animal-derived products, and the widespread development of acaricide resistance in tick populations [24,28,29].

The increasing resistance of *R.* (*B.*) *microplus* to conventional acaricides has stimulated the search for sustainable alternatives with lower environmental impact and reduced risk of resistance development [24,28,29]. In this context, coumarins have emerged as promising candidates for tick control due to their reported acaricidal and antiparasitic activities. Several studies have demonstrated the acaricidal potential of coumarin-rich plant extracts, as well as naturally occurring and hydroxylated coumarins such as scopoletin, against species of the genus *Rhipicephalus* and other arthropod pests [18,19,20,30,31]. However, most of these investigations have focused on natural products or a limited number of coumarin derivatives, providing insufficient information regarding the influence of structural modifications on acaricidal activity.

Furthermore, systematic evaluations of structurally diverse synthetic α-coumarin derivatives against *R.* (*B.*) *microplus* remain scarce. Likewise, few studies have integrated biological assays with computational approaches capable of elucidating structure–activity relationships and potential molecular mechanisms associated with acaricidal effects. Consequently, important knowledge gaps still exist regarding the structural features responsible for the biological activity of coumarin derivatives against this economically important tick species.

Therefore, the present study investigated the acaricidal activity of a series of α-coumarin derivatives synthesized through a Pechmann reaction using eco-compatible methodologies. Unlike previous studies focused mainly on coumarin-rich plant extracts or naturally occurring coumarins [18,19,20,30,31], this work evaluates a structurally diverse set of synthetic derivatives and integrates acaricidal bioassays with molecular docking and QSAR analyses. The purpose of the QSAR study is now described as identifying a statistically predictive descriptor and exploring structural correlates through virtual screening of new structures, rather than directly deriving mechanistic conclusions from the descriptor itself.

**Figure 1:**
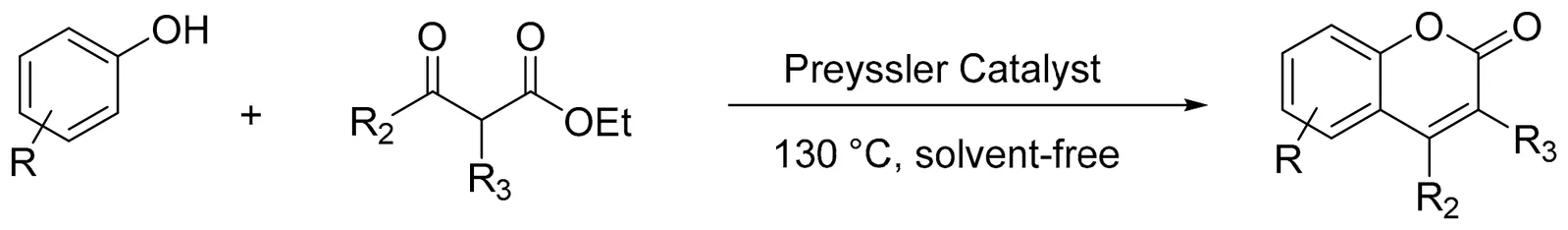
Pechmann reaction using Preyssler catalyst.

This integrated approach provides new insights into the structural determinants associated with acaricidal activity and contributes to the rational design of environmentally friendly alternatives for the control of *R.* (*B.*) *microplus*. The Pechmann reaction used for the synthesis of the coumarin derivatives is shown in Figure 1.

## 2. Results and Discussion

The procedure described above provides a useful, clean, and fast alternative for the preparation of coumarins 1–9, obtaining 25–95% yield of pure product (Table 1). The reaction time could be reduced 7–8 times compared to the classical methods. The importance of using this type of acid solid in this synthesis is the replacement of the corrosive acids conventionally used in the Pechmann reaction, such as concentrated sulfuric acid, trifluoroacetic acid, phosphorus pentoxide, aluminum chloride and ionic liquids, as catalysts. They require long reaction times and give rise to the formation of by-products and salt wastes due to the neutralization of the acid, limiting its reuse and generating unsatisfactory yields [32,33], unlike the heteropolyacids whose main benefit is the reduction in waste production, as well as their possible reuse.

Furthermore, coumarins 8 and 9 bear O-allyl and O-cinnamyl ether substituents at C7, rather than the free phenolic hydroxyl or simple alkyl/halomethyl groups present in compounds **1**–**6**. Pechmann condensation requires a free, nucleophilic phenolic hydroxyl positioned to enable cyclodehydration with the β-ketoester and subsequent lactone-ring formation; a phenol pre-alkylated at this position cannot undergo this cyclization. In addition, the strongly acidic, high-temperature conditions used in the Pechmann reaction would be expected to cleave the allylic and cinnamylic ether linkages, or promote their Claisen rearrangement, making direct one-step synthesis unsuitable for introducing these substituents. For this reason, compounds **8** and **9** were instead obtained through a two-step approach: first preparing the free C7-hydroxycoumarin (compound **1**) by the standard Pechmann reaction, then selectively O-alkylating its phenolic hydroxyl with allyl bromide or cinnamyl bromide under mild basic conditions (K_2_CO_3_/acetone), which leaves the pre-formed lactone ring untouched.

To identify the variables influencing the survival of *R.* (*B.*) *microplus* adults following exposure to each synthesized coumarin, a Cox proportional hazard regression model was fitted. The model evaluated the effects of treatment, exposure time, and dose on the instantaneous risk of death. In Table 2, hazard ratios (HR = expβ) were calculated for each explanatory variable using ethanol as the reference treatment. An HR greater than 1 indicates a higher instantaneous risk of death (reduced survival) relative to the reference group, whereas an HR lower than 1 indicates a lower risk. The final model was selected using likelihood ratio tests, showing that survival time was the primary factor explaining variation in mortality, while dose made a smaller but significant contribution.

The Cox proportional hazard model for the control treatments (Table 2) used ethanol as the reference group and compared its survival profile with those of distilled water and Ethion™. Relative to ethanol, the Ethion™ treatment exhibited a hazard ratio of 2.3, indicating a 2.3-fold higher instantaneous risk of death. In contrast, distilled water showed a hazard ratio close to 1, indicating a survival profile comparable to that of ethanol. These findings demonstrate that ethanol, used as the solvent for the synthesized coumarins, did not significantly affect tick survival and is therefore an appropriate vehicle control. Although Ethion™ produced a higher mortality than ethanol, its limited effect is consistent with the reported resistance of the Montecitos strain to organophosphate acaricides, as previously described in the literature.

Regarding the amount of coumarins used, it was found that the dose of 8 mg/mL presented the least main effect on engorged females; therefore, the model took this dose as a reference point to carry out the comparison of the main effects of the other doses, finding that in the case of the coumarins evaluated there is not always a direct relationship between the amount of molecule used and the decrease in survival, since although there is a greater decrease in survival as the dose is increased, the highest dose did not always present a greater main effect, as observed in the 10 mg/mL dose, which was superior to the 14 and 13 mg/mL doses (Table 3).

According to the limits of the confidence interval (Table 3), there are similarities between some of the doses evaluated, since according to Cumming et al. (2007) [34], it is possible to use 95% confidence intervals in the measurements, since the smaller the gap between these values is, the smaller the *p* value will be and the stronger the evidence of a true difference; according to this, doses represented with the same letters did not present significant differences between them, unlike with the rest of the doses.

The behavior of survival related to the doses used during the evaluation is explained by a phenomenon known as hormesis, since, although for dose–response relationships in a finite population subjected to a treatment in which the dose is increased proportionally, it is generally stated as a model that takes the form of the classic sigmoid curve, that is, as the dose increases, survival decreases [35], it can be stated that the “dose–response” relationship should not be taken as a result that counteracts or neutralizes the toxicity; rather, this type of test indicates the general effect that a certain treatment produces on an individual or group of individuals. Therefore, the hormetic response varies from the classic result, since it corresponds to biphasic behaviors that at low concentrations produce beneficial effects, while at high doses they cause detrimental effects [35]. When talking about pest management, these “beneficial” effects for the organism are considered as one of the probable causes of the resurgence and appearance of new pests, which is combined with sublethal exposures of pesticides that cause oxidative stress, which in turn produces an increase in the rate of change and in the activity of the detoxifying system, which could lead to the development of resistance [36,37].

An accession was used that has been recognized for its multiresistance characteristics, which agrees with the results obtained in this study given the low control obtained by the positive control (Ethion™), as well as the hormetic behavior of the response.

The Cox proportional hazard model applied to each synthetized coumarin allows us to compare the interactions presented by the variables considered (time and dose), comparing the main effects of coumarin 1, which presented the least effect. With respect to the rest of the coumarins evaluated, however, using the values of the limits of the confidence interval (Table 4) it was possible to show similarities between some of the treatments evaluated. Coumarin 4 presented a main effect 5.7 times greater when compared to coumarin 1; in addition, it did not present similarities with other coumarins, since its confidence intervals did not overlap with those of any other treatment, and therefore it was considered that this coumarin has the greatest acaricidal effect.

Coumarins 8 and 3 have a main effect 3.1 and 2.6 times greater than that presented by coumarin 1. Although these have a difference of 0.5 points with respect to their main effect, the limits of the confidence interval show that there are similarities between their effect on the survival of *R.* (*B.*) *microplus*. Similar results were found with coumarin 7, which exhibited main effects 2.2 times higher than coumarin 1; however, according to the confidence intervals, they presented similarities with coumarin 3, but not with coumarin 8 (Table 4).

With respect to coumarins 2, 5 and 9, the main effects were between 1.17 and 1.4 times higher than that produced by coumarin 1, so they formed a group with a medium effect on the survival of *R.* (*B.*) *microplus* adults.

The results of the main effects of the synthetized coumarins allow us to appreciate that the variations in their structure caused a greater or lesser effect on the survival of engorged females of *R.* (*B.*) *microplus*, and the differences in these values could be attributed to a different location of the substituents in the scaffold of the coumarins (Table 1), which increased their additional interactions and the substitution pattern determined, increasing the energy and power of the general union and therefore the specificity of the molecules [1]. Comparing these results with the IC_50_ values obtained by Probit analysis of the mortality of engorged females (Table 5), it resulted that examining the only pair that differs solely at C3 (compounds **1** and **3**: R3 = H vs. CH_3_, all other substituents identical), the IC_50_ decreases from 14.14 to 10.52 mg/mL, indicating that C3-methylation actually increased activity in this specific comparison. On the other hand, compounds **1**, **8**, and **9** share identical substituents at C3, C4, and C5, differing only at C7 (OH, allyloxy, and cinnamyloxy, respectively), with IC_50_ values of 14.14, 7.24, and 10.94 mg/mL. This direct comparison shows that a moderately sized aliphatic ether at C7 (compound **8**) is the most favorable, while extending the chain with an aromatic group (compound **9**) reduces activity relative to compound **8**, though it remains more active than the C7-OH parent (compound **1**). The hydroxyl groups on the α-pyrone ring of coumarins result in adequate bioactive principles for tick control. Scopoletin is the most studied hydroxycoumarin in the control of insects [19,20]. The position in C_6_ with the methoxy group in C_5_ showed greater activity than other coumarin derivatives in the control of *Protium javanicum* [20]. However, the hydroxyl position in C_6_, C_7_ showed a high mortality in this same species. 4-Hydroxycoumarins present a significant attractive effect on *Anopheles* spp. and significant selectivity towards *Anopheles gambiae s.l.* and *Anopheles mascarensis,* which are both significant malaria vectors in Madagascar [30,31]. In addition, it has been reported that the mortalities of the larvae of *Rhipicephalus appendiculatus* showed an IC_50_ near to 3 mg/mL when the extract contained 8-hydroxycoumarin as the principal bioactive component [18].

As the substitution of the hydroxyl group is mainly responsible for the biological activity, among distinct targets we chose triosephosphate isomerase (TIM), since it is commonly used to understand the acaricidal effect [38,39,40]. TIM was selected as the docking target based on its established role in tick energy metabolism (both glycolysis and gluconeogenesis), the availability of a crystallographic structure of the recombinant enzyme from *R.* (*B.*) *microplus* (PDB 3TH6) [38], and prior reports demonstrating that selective inhibitors acting at its poorly conserved dimer interface can suppress tick metabolism and exert acaricidal effects [39,40]. Other recognized acaricide targets, such as acetylcholinesterase, GABA-gated chloride channels, octopamine receptors, and voltage-gated sodium channels, are well characterized for synthetic neurotoxic acaricides [41], but were outside the scope of the present exploratory docking study.

**Figure 2:**
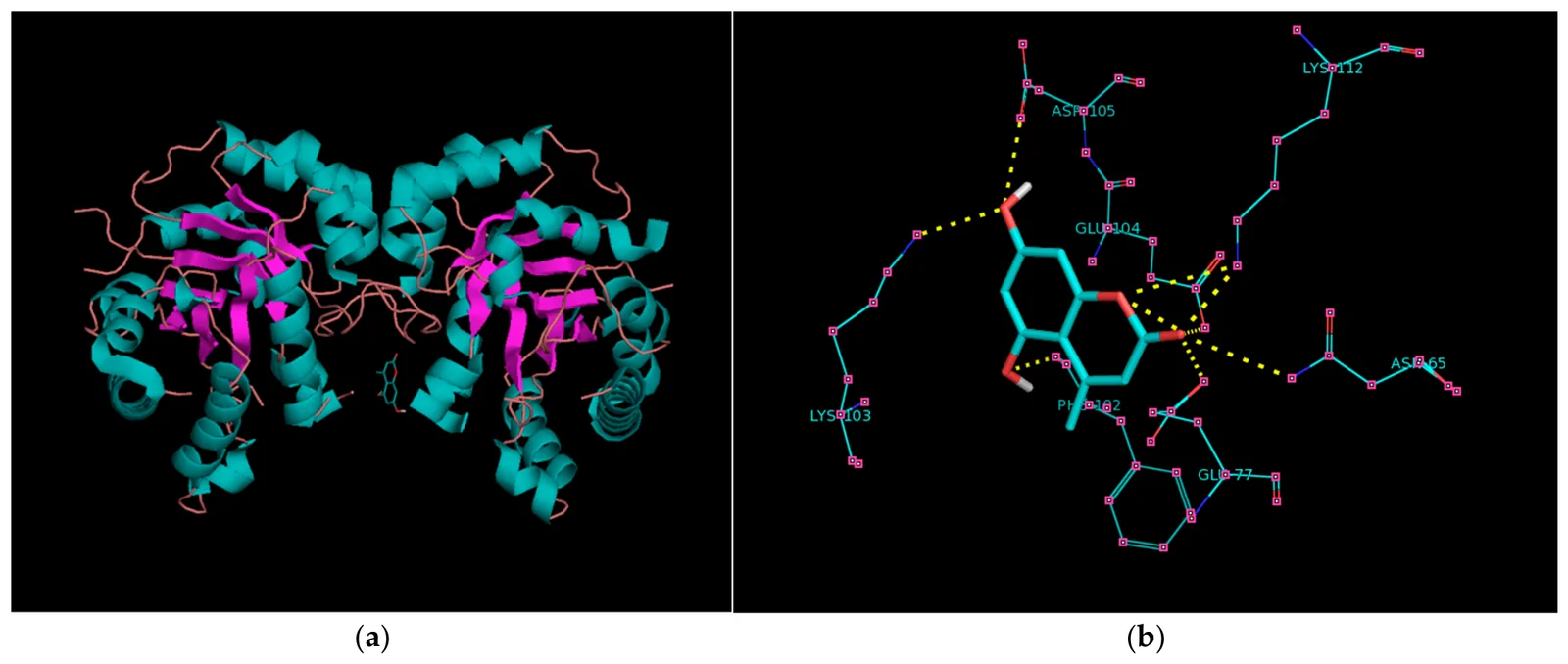
(**a**) Best pose of 5,7-dihydroxy-4-methyl-2H-chromen-2-one along the tunnel-shaped cavity at the interface of TIM. (**b**) Interaction scheme of 5,7-dihydroxy-4-methyl-2H-chromen-2-one in TIM. (**a**) TIM structure showing the two monomers in cyan and magenta. (**b**) Detailed view of the binding interactions between the ligand (cyan) and the active-site residues. Hydrogen-bond interactions are represented by yellow dashed lines.

Table 5 displays the IC_50_ values, energy of interaction, and the amino acid residues that participate in the interaction of TIM–coumarins. It is well known that the binding energies are not correlated with biological activity, but the best pose could give an account of the interaction of each compound in the protein, given TIM’s established role as a validated and selectively druggable target in *R.* (*B.*) *microplus* [38], the demonstration that selective inhibitors acting at the dimer interface suppress tick energy metabolism [39,40], and the structural conservation of this interface cavity across related parasitic organisms [41,42]. Compounds **2** and **4** with better IC_50_ values tend to lie along the tunnel-shaped cavity at the interface formed by the residues (Figure 2), whereas the non-inhibitory coumarins 1 and 5 do not choose this region. In the interface, the residues Lys-112, Asn-65, and Glu 77 interact with the lactone carbonyl group of coumarin, as can be observed in compounds **2** to **4**. Coumarin 8 also chooses this region, but a hydrophobic substituent in C_7_ modifies the interaction with these residues, and this compound penetrates better into the interface of the tunnel of TIM. Thus, the IC_50_ values could be rationalized using the best pose of coumarins in TIM. This behavior has been broadly described to explain the inhibitory effect on TIM of benzothiazoles against *Trypanosoma cruzi* [42]. In fact, this corroborates the importance of the tunnel of TIM for the inhibition process of this type of parasite.

As illustrated by compound 9, which displays the most favorable calculated binding energy in the series but is not the most experimentally active compound, binding energy alone does not reliably predict acaricidal potency in this dataset. This is consistent with the well-established limitation that docking scores, which estimate the enthalpic contribution of a single static pose, do not capture factors such as compound solubility, membrane or cuticle penetration, metabolic stability, or off-target interactions, all of which influence activity in a whole-organism bioassay.

The quantitative structure–activity relationship was built by searching for the best molecular descriptor among the 60,642 available non-conformational structural variables. In this way, the smallest value for *RMSE* in the training set RMSE_train_ was achieved for predicting the logarithmic IC_50_ acaricidal activity:

logIC_50_ = 5.002d_1_ − 3.304 (1)

N _train_ = 7, R^2^_train_ = 0.79, RMSEtrain = 0.051,   o2 = 0

R^2^_loo_ = 0.80, RMSE_loo_ = 0.061,  R^2^_rand_ = 0.172, RMSE_rand_ = 0.100, R^2^_m_ = 0.88

N _test_ = 2, RMSE _test_ = 0.059

where d1 stands for 5CN-NE(DE)-E-WH-T, a topological descriptor derived from theory through MD-LOVIs. As appreciated, the structure–activity relationship between this single descriptor and IC_50_ was quite direct: the lower the numerical value of d_1_, the higher the predicted acaricidal activity (lower IC_50_ predicted) for the analyzed coumarin derivatives. 

The d_1_ index was obtained as a total (global) descriptor that considered all the substructure contributions, while the hydrogen-filled graph representation was used together with Pauling’s electronegativity characterizing heteroatoms. The contributions from the Kier–Hall connectivity operator of order 5 were first derived, while the standard deviation was finally used as an aggregation operator of these non-standardized LOVIs.

Although the chemical interpretation of this topological descriptor remained hidden behind the algebraic definition, it was important for predicting the acaricidal activity of coumarin derivatives. This descriptor is a single number that summarizes, for each coumarin molecule, how its atoms are connected to one another and how electron-attracting its heteroatoms (such as oxygen) are. The value reported here is calculated using their statistical spread (standard deviation), which captures how much these local atomic properties vary across the molecule. Molecules with a lower value of this descriptor are predicted, by the model, to have stronger acaricidal activity [43].

**Figure 3:**
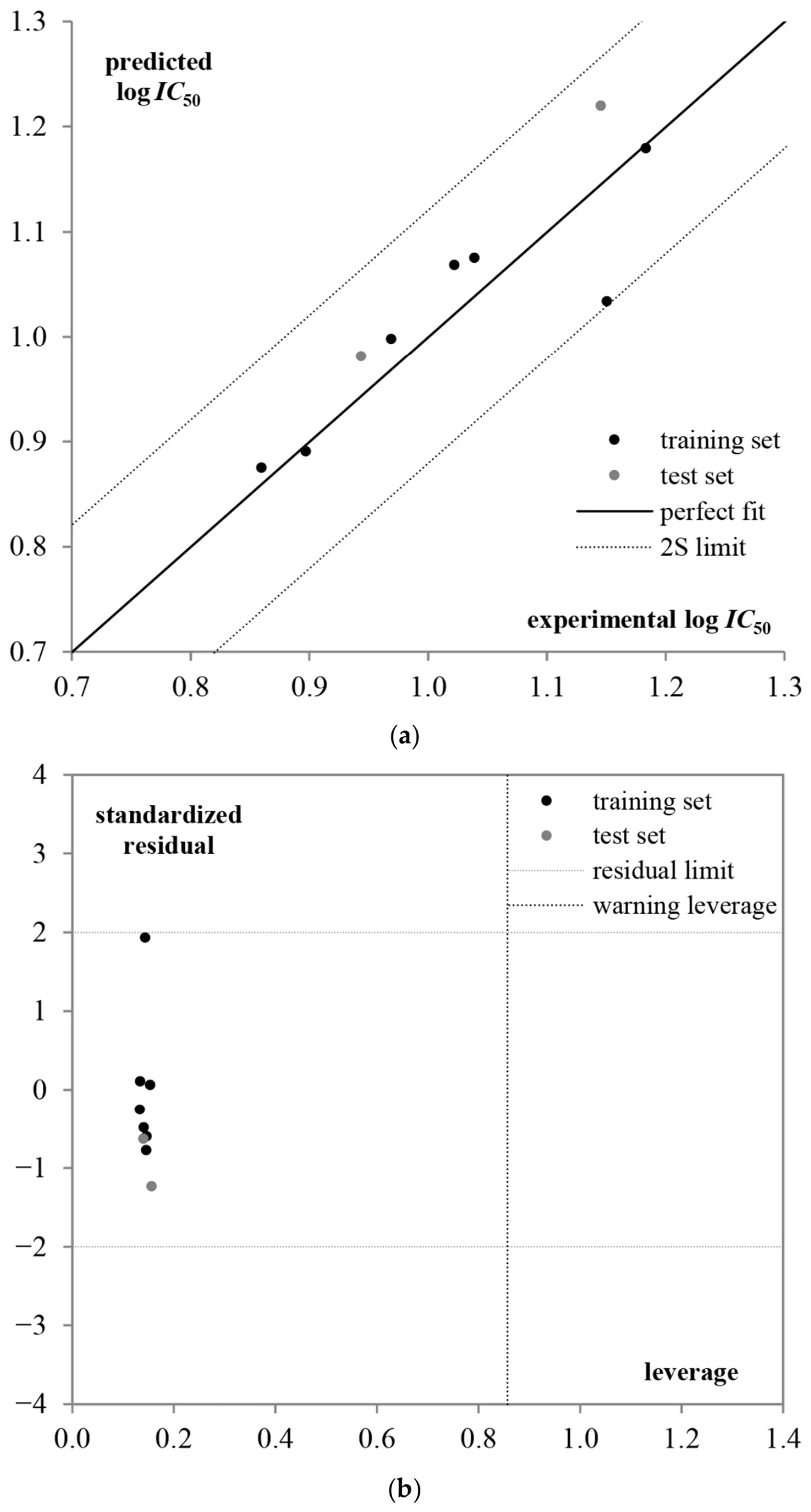
(**a**) Experimental and QSAR predicted acaricidal activity of coumarins. (**b**) Standardized residual as a function of leverage.

Equation (1) did not involve outlier training set coumarins with high residuals: the number of compounds posing residuals greater than two times S train (o2) was zero. A plot for the log IC_50_ prediction by Equation (1) as a function of the experimental value is shown in Figure 3a. Acceptable predicted bioactivities were found for the two test set coumarins not considered during the model calibration. This plot revealed a straight line trend for all the modeled data. Table S1 includes such predictions for the nine acaricidal coumarins, indicating the molecules that were selected as part of the test set according to the BSM technique. Table S2 lists the numerical values for d1.

The reliability of the QSAR predictions could be estimated through the model’s applicability domain (AD), a theoretically defined space that contains only molecules not considered as model extrapolations [34,44]. Figure 3b plots the standardized residual of Equation (1) as a function of the leverage value, revealing that the nine modeled compounds belong to the AD, with leverage values falling under the warning leverage limit of 0.857.

The theoretical validation process of the leave-one-out (loo) cross-validation technique [45] succeeded in predicting one molecule excluded at a time from the training set, achieving R^2^_loo_ > 0.5 and thus revealing the stability of Equation (1). The R^2^_m_ index [46] was also acceptable for Equation (1), with R^2^_m_ > 0.5.

It was possible to demonstrate that Equation (1) did not involve chance correlation through Y-randomization [47], as R^2^_rand_ < R^2^_train_ and RSME_rand_ and > RMSE_train_. In this assay, the activity value was randomized (scrambled) for each analyzed case, while maintaining the selected descriptor (the regression coefficients were recomputed). The number of tested cases was 100,000, and the average R^2^_rand_ and RMSE_rand_ values were reported.

The ultimate goal of any QSAR study is to apply the so-established quantitative relationship for predicting new structurally related compounds having unknown experimental activity. In this work, an improvement of the acaricidal activity of the nine coumarins synthesized in the laboratory was searched, by making small structural modifications in the coumarin unit.

The QSAR model was built on a small dataset of nine compounds, with only seven in the training set and two in the external test set. A test set of this size cannot, on its own, provide a statistically robust estimate of true external predictive performance, and we have revised our language throughout the manuscript to avoid overstating the model’s predictive power. The validation steps performed, leave-one-out cross-validation, Y-randomization over 100,000 trials, the rm2 metric, and applicability domain analysis, are intended to guard against chance correlation given the limited sample, but they do not substitute for validation on a larger, independent set of compounds. The QSAR model should therefore be regarded as a preliminary, exploratory tool for prioritizing candidate structures for future synthesis, rather than as a fully validated predictive model.

By observing the experimental results, it was noted that the two coumarins that presented the highest activity were 8 IC_50_[mgml^−1^] = 7.24 and **2** (IC_50_[mgml^−1^] = 7.89). In the case of coumarin 8, the presence of an unsaturated side chain of three carbon atoms would increase the activity of this family of compounds. On the other hand, in the case of coumarin 2 the presence of -OH groups, particularly in positions 5 and 7, would produce the same effect.

Therefore, in order to validate the QSAR model obtained, 41 examples of new coumarin structures were postulated, which could be obtained in the future by simple and low-cost organic synthesis methods. Table S1 also includes the predictions found for these 41 new coumarin derivatives according to Equation (1). All of these compounds fell within the AD and had leverages under the warning leverage limit: their predictions could be considered as reliable. The bioactivity prediction confirmed our suspicions, obtaining fourteen structures (31, 50, 29, 49 30, 42, 21, 48, 44, 32, 43, 28, 27, and 47) that were predicted to be more active than coumarin 8.

By analyzing the molecular structure of the coumarin predicted as the most active by the proposed model (**31**), it was confirmed that the presence of -OH groups increased the activity; this was verified by the incorporation of an -OH group in C_4_ methyl. There is ample evidence in the literature that the presence of hydroxyl groups in coumarins notably increases their bioactivity. On the other hand, a second effect could be associated with the length of the chain in C_7_. From the analysis of the postulated structures, it was found that the structures having a side chain of five carbon atoms are the most active (31 and 50), reducing the activity in chains of greater and shorter length. Likewise, the effect associated with the presence of a double bond in it was not so relevant, confirming a priori that the effect would be mostly associated with the polarity of the hydrocarbon substituent.

## 3. Materials and Methods

### 3.1. Materials and Methods

Chemicals were purchased from Aldrich (Milwaukee, WI, USA) and Fluka (Buchs, Switzerland) and purified immediately before use by standard procedures (distillation or recrystallization). Reactions were monitored by thin-layer chromatography (TLC) on precoated silica gel plates (254 mm). When purification of the product was necessary, flash chromatography was performed using 230–400 mesh silica gel. (Silica gel Inorganic Sorbent, Aldrich-Argentina, Buenos Aires, Argentina).

### 3.2. Catalyst Preparation

The catalyst H_14_(NaP_5_W_29_MoO_110_) was synthesized according to a procedure reported by our research group [48], by following a procedure analogous to that used for the preparation of Preyssler heteropolyacid (HPA). Initially, Na_2_WO_4_·2H_2_O (23 g, 0.07 mol) and Na_2_MoO_4_·2H_2_O (2 g, 8.3 mmol) were dissolved in 20 mL of water and stirred at 333 K for 30 min. Subsequently, 27 mL of H_3_PO_4_ was added, and the reaction mixture was refluxed for 24 h. After cooling to room temperature, a solution of KCl (10 g, 0.13 mol) in 30 mL of water was introduced under vigorous stirring for an additional 30 min. The resulting precipitate was recrystallized from 70 mL of warm water and allowed to cool to room temperature, yielding yellow crystals of K_14_[NaP_5_W_29_MoO_110_]. Finally, the potassium salt was converted into its protonated form, H_14_[NaP_5_W_29_MoO_110_], by passing it through a column packed with Dowex-50W-X8 cation-exchange resin.

### 3.3. Coumarin Synthesis

Coumarins 1-6 were synthesized using green chemistry methodologies [49,50] by a Pechmann reaction from a mixture of the appropriate phenol (15 mmol) and the corresponding ethyl acetoacetate derivative (15 mmol) under solvent-free conditions. Phenol and 3-phenylpropiolic acid were used in the same proportions to synthetize the coumarin 7. For all syntheses, the reactants were mixed and maintained at 130 °C under constant agitation for 60 min in the presence of bulk H_14_NaP_5_W_30_O_110_ Preyssler heteropolyacid (1 mol%). Reactions were performed in a round-bottom flask equipped with a condenser and heated in an oil bath. After cooling, the mixtures were extracted twice with hot toluene (7 mL each). The solvent was subsequently removed to afford the crude products, which were purified through recrystallization using methanol or ethanol as the crystallization solvent.

The O-alkylation of the hydroxyl group in C7 of compound **1** was carried out in the presence of K_2_CO_3_ using 7 mmol of allyl bromide and cinnamyl bromide to obtain coumarins 8 and 9, respectively. Briefly, 5 mmol of compound **1** was refluxed with 1 mmol of K_2_CO_3_ in 50 mL of acetone for 1 h. To this dilution, 7 mmol of bromide derivative was added and then heated to reflux for approximately 4 h. The solution was concentrated under vacuum, and the product was extracted with dichloromethane (3 × 10 mL), dried with Na_2_SO_4_, and then recrystallized from methanol.

Yields were calculated for crystallized products, which were identified by comparing their analytical data (melting point, TLC, and NMR) with literature values or with authentic samples prepared by the conventional method using sulfuric acid as a catalyst. Melting points were determined in sealed capillary tubes and are uncorrected. ^13^C NMR and ^1^H NMR spectra were recorded at 20 °C on a Bruker AC-400 spectrometer (100 and 400 MHz, respectively, Bruker BioSpin GmbH, Karlsruhe, Germany), using TMS as an internal standard. NMR data acquisition and primary processing were performed using XWIN-NMR software version 2.6 (Bruker BioSpin, Rheinstetten, Germany).

### 3.4. Product Characterization of Selected Coumarins

Compound **1**: 7-hydroxy-4-methyl-2*H*-chromen-2-one. mp 186–188 °C (methanol). ^13^C-NMR (DMSO-d_6_, 100 MHz): δ 162.6, 161.7, 155.4, 155.0, 127.5, 114.1, 113.2, 110.8, 103.1, 18.9. ^1^H-NMR (DMSO-d_6_, 400 MHz): δ 10.55 (1H, s), 7.44 (1H, d, *J* = 8.8 Hz), 6.70 (1H, dd, *J* = 8.8 Hz, *J* = 2.3 Hz), 6.57 (1H, d, J = 2.3 Hz), 5.97 (1H, d, J = 1.1 Hz), 2.23 (3H, d, J = 1.0 Hz).

Compound **2**: 5,7-dihydroxy-4-methyl-2H-chromen-2-one. mp 285–287 °C (methanol) ^13^C-NMR (DMSO-d_6_, 100 MHz): δ 162.5, 161.7, 159.5, 158.8, 157.2, 109.5, 103.5, 100.3, 95.6, 24.3. ^1^H-NMR (DMSO-d_6_, 400 MHz): δ 10.54 (1H, s), 10.31 (1H, s), 6.26 (1H, d, *J* = 2.2 Hz), 6.16 (1H, d, *J* = 2.2 Hz), 5.85 (1H, d, *J* = 1.1 Hz), 2.49 (3H, d, *J* = 0.9 Hz).

Compound **3**: 7-hydroxy-3,4-dimethyl-2H-chromen-2-one. mp 255–256 °C (methanol). ^13^C-NMR (DMSO-d_6_, 62.5 MHz): δ 161.1, 159.8, 152.9, 146.6, 125.9, 116.7, 112.5, 112.3, 101.7, 14.5, 12.5. ^1^H-NMR (DMSO-d_6_, 250 MHz): δ 10.35 (1H, s), 7.54 (1H, d, *J* = 8.5 Hz), 6.75 (1H, dd, *J* = 8.5, *J* = 2.1 Hz), 6.65 (1H, d, *J* = 2.1 Hz), 2.29 (3H, s), 2.02 (3H, s).

Compound **4**: 5-hydroxy-3,4,7-trimethyl-2H-chromen-2-one. mp 249–251 °C (methanol) ^13^C-NMR (DMSO-d_6_, 100 MHz): δ 159.7, 156.4, 154.8, 154.5, 142.7, 111.9, 107.6, 107.0, 106.5, 23.4, 21.0. ^1^H-NMR (DMSO-d_6_, 400 MHz): δ 10.5 (1H, s), 6.62 (1H, s), 6.58 (1H, s), 6.05 (1H, s), 2.54 (3H, s), 2.28 (3H, s).

Compound **5**: 4-(chloromethyl)-7-hydroxy-2H-chromen-2-one. mp 172–173 °C (methanol). ^13^C-NMR (DMSO-d_6_, 100 MHz): δ 165.0, 160.9, 160.1, 156.9, 148.6, 117.3, 117.2, 113.1, 109.5, 50.2, 26.4. ^1^H-NMR (DMSO-d_6_, 400 MHz): δ 10.92 (1H, s), 6.68 (1H, s), 6.60 (1H, s), 6.41 (1H, s), 5.08 (2H, s), 2.29 (3H, s).

Compound **6**: 4-(chloromethyl)-5-hydroxy-7-methyl-2H-chromen-2-one. mp 220–221°C (methanol) ^13^C-NMR (DMSO-d_6_, 100 MHz): δ159.7, 155.6, 154.7, 151.5, 143.2, 112.0, 111.7, 107.8, 104.2, 45.0, 21.1. ^1^H-NMR (DMSO-d_6_, 400 MHz): δ 10.90 (1H, s), 6.65 (1H, s), 6.60 (1H, s), 6.40 (1H, s), 5.07 (2H, s), 2.29 (3H, s).

Compound **7**: 4-phenyl-2H-chromen-2-one. mp 102–104 °C (methanol). ^13^C-NMR (CDCl_3_,100 MHz), δ 162.1, 159.2, 148.0, 135.0, 128.1, 127.8, 127.5, 126.6, 126.2, 126.1, 125.1, 121.3, 106.0. ^1^H-NMR (CDCl_3_, 400 MHz,), δ 7.5 (2H, dd, *J* = 2.5 Hz, *J* = 8.0 Hz), 7.33 (5H, m), 7.2 (2H, dd, *J* = 2.5 Hz, *J* = 8.0 Hz), 6.42 (1H, s).

Compound **8**: 4-methyl-7-(prop-2-en-1-yloxy)-2H-chromen-2-one. mp 100–101 °C (methanol). ^13^C-NMR (CDCl_3_, 100 MHz), δ 161.8, 161.5, 155.4, 152.8, 132.4, 125.7, 118.7, 113.9, 113.0, 112.2, 102.0, 69.4, 18.8. ^1^H-NMR (CDCl_3_,400 MHz), δ 7.44 (2H, d, *J* = 16.0 Hz), 6.10 (2H, s), 5.90–6.09 (2H, m), 5.25 (1H, d, *J* = 12.0 Hz), 4.54 (2H, d, *J* = 12.0 Hz), 2.34 (3H, s).

Compound **9**: 4-methyl-7-{[(2E)-3-phenylprop-2-en-1-yl]oxy}-2H-chromen-2-one. mp 179–181 °C (methanol). ^13^C-NMR (CDCl_3,_100 MHz), δ 161.8, 161.5, 155.5, 152.8, 136.3, 134.2, 128.9, 128.4, 126.7, 125.8, 123.3, 118.9, 113.0, 112.3, 102.0, 69.4, 18.9. ^1^H-NMRM (CDCl_3_, 400 MHz), δ 7.26–7.52 (6H, m), 6.72–6.94 (2H, m), 6.32–6.45 (2H, m), 6.12 (1H, s), 4.75 (2H, d, *J* = 16.0 Hz), 2.38 (3H, s).

### 3.5. Acaricidal Activity

The acaricidal activity of coumarins on *R.* (*B.*) *microplus* was determined using the immersion test described by Drummond et al., [51] and adapted by Ghosh et al., [52] known as the adult immersion test. Ticks were obtained from the experimental center Tibaitatá (Agrosavia) and, prior to the test, they were washed with distilled water for 5 min and then dried with absorbent paper.

For each evaluated compound, five groups of ten females were formed, which were selected according their weight (average 270 mg) and wholeness so that each group was homogeneous. In addition to the nine evaluated coumarins, two negative controls (distilled water and absolute ethanol) and a positive control (ethion 83%) were made, the latter according to commercial preparation recommendations.

Once the groups were formed, they were immersed for 5 min in each of the coumarin concentrations, which ranged from 15 mg/mL to 8 mg/mL. After the immersion, individuals were dried with absorbent paper and placed in labeled Petri dishes, which were stored in a biochemical oxygen demand incubator at 27 ± 1 °C and relative humidity ≥ 80% [53].

The mortality behavior of each group was observed daily for a period of 4 days or until the death of the group, since generally the effects produced by substances for the control of ticks do not occur immediately, and the symptoms of the treatments are observed after this period. Assessment of the engorged females was carried out using a stereoscope to facilitate the observation of the number of dead ticks, which are recognized for presenting lesions on the cuticle or darkening of the cuticle, in addition to the lack of movement of the legs or displacements in response to different stimuli such as a light source (stereoscope lamp) or the reaction to being touched with a blunt pointer in the cervical region.

### 3.6. Statistical Analysis

IC_50_ values were estimated from the dose–response equations generated for each coumarin derivative. Eleven statistical models, including log-logistic, Weibull, Probit, and third-degree polynomial models, were fitted to the mortality data [54]. The best-fitting model was selected according to the Akaike Information Criterion (AIC). Subsequently, IC_50_ values were determined from the selected model using a numerical optimization procedure implemented with the Solver tool in Microsoft Excel. Statistical analyses were performed using R software (version 3.6.3) with the packages drc and survival [54,55].

The relationship between coumarin treatment, exposure time, and survival of engorged females was evaluated using the Cox proportional hazard model [56,57]. This approach allows the estimation of survival probabilities over time while accounting for censored observations, thereby providing a more reliable assessment of the instantaneous risk associated with each treatment. The model was used to compare the hazard of reaching complete mortality among females exposed to different coumarins and concentrations, enabling evaluation of the main effects of each compound under study.

For coumarins 2 and 9, concentrations of 13, 14, and 15 mg/mL were excluded from the analysis because complete mortality of *R.* (*B.*) *microplus* had already been observed at 10 mg/mL. Such observations were treated as censored data, corresponding to outcomes that could not be further evaluated within the predefined experimental period due to the occurrence of the event of interest before completion of all treatment levels.

### 3.7. Ligand–Protein Molecular Docking

Optimized structures were performed using the B3LYP/6–31^+ +G^** level using the Gaussian 09 software [58]. For molecular docking studies, the crystallographic structure of triosephosphate isomerase (RmTIM, PDB ID 3TH6) was used [38]. Docking calculations were carried out with AutoDock 4.2 using the Lamarckian genetic algorithm. The interaction of coumarin–residues was explored on the entire protein surface using a grid map with 124 × 126 × 126 points and a grid-point spacing of 0.6 Å using AutoDockTools software 4.2. PyMOL V0.99 viewer was used to draw the docking results.

### 3.8. Calculation of Molecular Descriptors and Removal of Collinear Variables

Different freely available and open-source software applications were combined for computing a large pool of molecular descriptors. In total, 98,490 conformation-independent descriptors were calculated for the coumarin derivatives, as described next.

Molecular descriptors were generated using several complementary software packages. Initially, the Pharmaceutical Data Exploration Laboratory (PaDEL) was employed to calculate 17,536 one- and two-dimensional molecular descriptors together with different fingerprint representations [59,60]. Molecular structures were provided in MDL mol (V2000) format, applying the options for nitro-group standardization and aromaticity detection. File conversion to MDL sdf format was performed using Open Babel for Windows [61].

Additional descriptor sets were obtained with Mold2, which generated 777 one- and two-dimensional descriptors [62]. Structural fragment information was derived using ISIDA/Fragmentor [63,64], considering substructures containing between one and six atoms, resulting in a set of 97 constitutional descriptors.

To further characterize the compounds, Molecular Descriptors from Local Vertex Invariants (MD-LOVIs, version 1.0) was used. Both global and fragment-based local indices were calculated. The atom properties considered included atomic number, van der Waals volume, polarizability, atomic mass, covalent radius, Pauling electronegativity, total polar surface area, AlogP, molar refractivity, and atomic charge. Several vertex-degree definitions were also incorporated, including valence degree, eccentric connectivity, electrotopological state, Kupchik, Li, Hu–Xu, Alikhanidi, and Ivanciuc indices, together with distance-count descriptors.

For local descriptors, the retained fragment classes comprised heteroatoms, carbon atoms, halogens, hydrogen-bond acceptors, hydrogen-bond donors, methyl groups, unsaturated bonds, aliphatic atoms, aromatic atoms, and group_lagk descriptors considering topological distances from 1 to 8. No standardized invariants were applied. Descriptor aggregation was performed using Euclidean-distance metrics, arithmetic means, standard deviations, autocorrelation functions, gravitational indices, Kier–Hall connectivity indices, total sum k-lags, information-content descriptors, electrotopological-state descriptors, and Ivanciuc–Balaban-type indices. Overall, the MD-LOVIs procedure generated a final set of 80,080 descriptors.

The 98,490 computed molecular descriptors were analyzed in order to eliminate collinear descriptors having redundant information. Thus, highly correlated descriptor pairs were identified, and only the most interpretable variable from each pair was maintained for further analysis. A data-cleaning step was subsequently performed to improve the quality of the descriptor set. Variables showing negligible information content, such as constant or almost invariant descriptors, as well as descriptors with incomplete data, were discarded. This preprocessing stage resulted in a reduced dataset comprising 60,642 molecular descriptors.

#### Model Development

When few experimental data were available, the empirical “rule of thumb” [65] indicated that more than six experimental observations per variable should be used during model development. With the purpose of avoiding overfitting problems and chance correlation, univariable linear regression models were established. The best descriptor was searched among the 60,642 available ones that led to the smallest value for the root mean square error (*RMSE*) or the standard deviation (*S*) in the training set.

The structure–acaricide activity relationship was constructed by searching for the best molecular descriptor among the available non-conformational structural variables. Likewise, the lowest value of the root mean square error (RMSE) error in the set of coumarins was used to predict the logarithmic acaricidal activity (IC_50_).

The dataset was divided into training and external test subsets using the Balanced Subsets Method (BSM) [66]. Seven compounds (78% of the dataset) were assigned to the training set and used for model development, whereas the remaining two compounds (22%) constituted an independent test set employed exclusively for external validation of the model’s predictive performance.

BSM was designed to generate representative subsets by preserving the structural and biological diversity of the original dataset. Unlike random sampling procedures, this approach aims to maintain comparable structure–activity relationships in both subsets. The method relies on k-means cluster analysis (k-MCA) [67], which groups compounds according to their similarity based on a selected distance metric—in this case Euclidean distance. As a result, compounds within the same cluster exhibit high similarity, whereas those belonging to different clusters are more dissimilar.

In addition to external validation using the test set, model robustness and predictive ability were evaluated through a series of theoretical validation criteria previously reported in the literature [45,46,47,68,69]. All algorithms implemented in GNU Octave for data analysis and model development were programmed in-house and can be provided upon request.

## 4. Conclusions

This study revealed the good behavior of the catalyst H_14_(NaP_5_MoW_29_O_110_) for the synthesis of coumarins under solvent-free conditions. This green protocol allows us to obtain coumarins with high yields in short reaction times. The proportional risk model displays the resistance of engorged females to the organophosphate Ethion. A greatest acaricidal effect was obtained using 5,7-dihydroxy-4-methylcoumarin, 5-hydroxy-3,4,7-trimethyl-coumarin, and 7-cinnamyloxy-4-methylcoumarin, which presented survival values equal to or close to 0% at a concentration of 10 mg/mL. The survival behavior of *R.* (*B.*) *microplus* decreased as the concentration of each coumarin increased. However, it was possible to appreciate a hormetic response for some of the coumarins which is related to variations in the functional group and its location within the structure. In general, the acaricidal effect of the synthesized coumarins demonstrates that hydroxycoumarins with hydroxyls in position C_5_ and C_7_ or with larger substituents in position C_7_ are good candidates against the adult cattle tick *R.* (*B.*) *microplus,* which was confirmed with QSAR studies and in part can be explained considering the binding mode on the tunnel of triosephosphate isomerase. Subsequent studies will be carried out in our laboratory on the synthesis of the coumarins that turned out to have greater bioactivity through procedures of low environmental impact. In addition, future work will focus on in vivo evaluation of the most active derivatives (5,7-dihydroxy-4-methylcoumarin, 5-hydroxy-3,4,7-trimethylcoumarin, and 7-cinnamyloxy-4-methylcoumarin) in order to confirm their acaricidal efficacy, assess potential toxicity to the host animal, and determine appropriate dosing under field-relevant conditions, as a step toward their potential application as alternatives to conventional acaricides.

## Figures and Tables

**Table 1:** Yields obtained from distinct coumarins using Preyssler heteropolyacid.

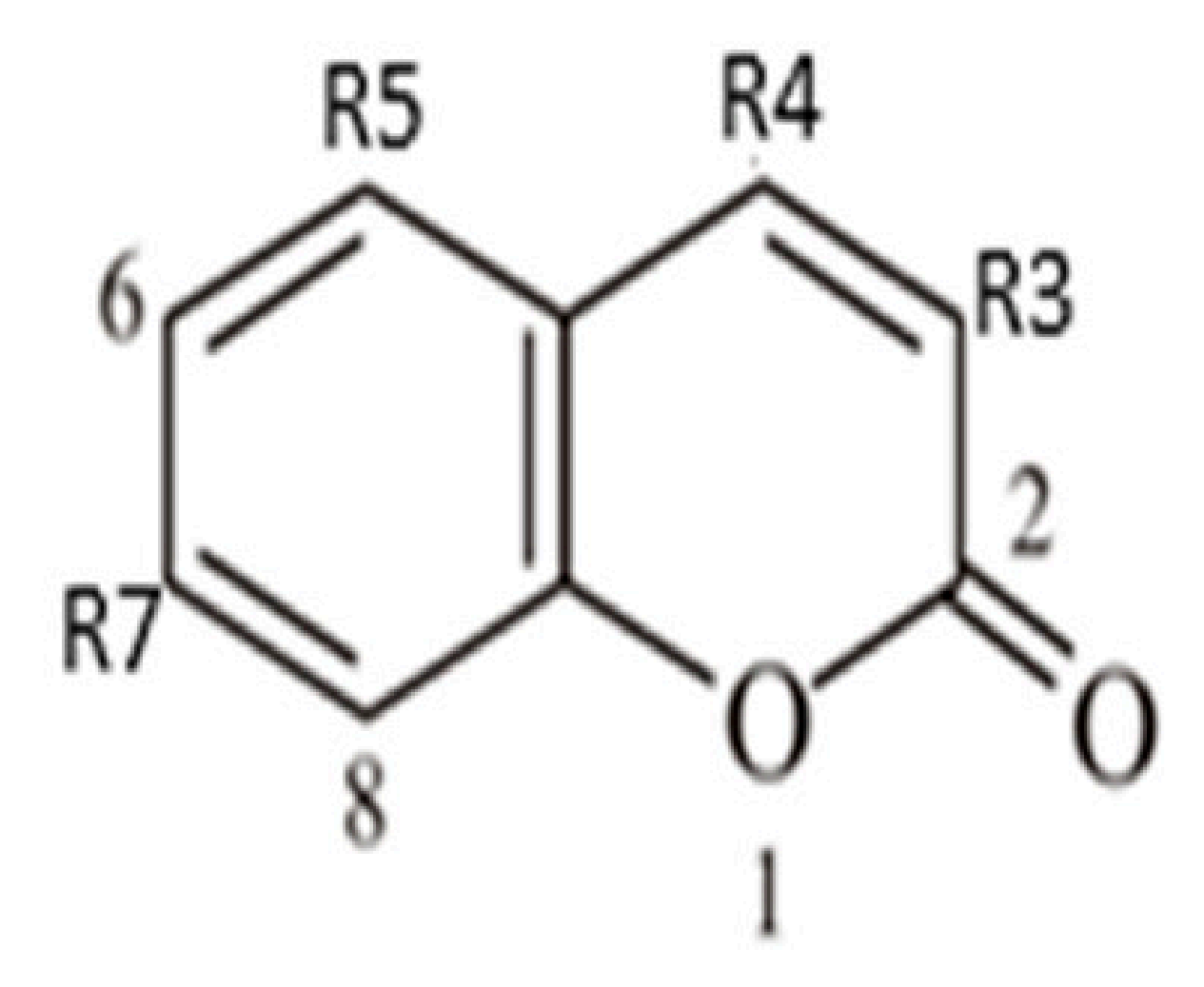					
Compound	R3	R4	R5	R7	Yield (%)
**1**	H	CH_3_	H	OH	88
**2**	H	CH_3_	OH	OH	69
**3**	CH_3_	CH_3_	H	OH	77
**4**	CH_3_	CH_3_	OH	CH3	75
**5**	H	CH_2_Cl	H	OH	75
**6**	H	CH_2_Cl	OH	CH3	69
**7**	H	Phenyl	H	H	95
**8**	H	CH_3_	H	3-methoxyprop-1-ene	83
**9**	H	CH_3_	H	3-phenylprop-2-en-1-yl	25

**Table 2:** Cox proportional hazard model applied to the controls.

Treatment	Coef	Se(coef)	Exp(coef)	Lower .95	Upper .95	Z	Pr(>|z|)	Sig.
Control H_2_O ^a^	0.00111	0.8165	1.00111	0.2021	4.96	0.001	0.999	0.001
Ethion™ ^b^	0.85683	0.69007	2.35568	0.6092	9.11	1.242	0.214	0.001

^a^ Distilled water (negative control). ^b^ Ethion™ 83% (positive control). Contrast of the estimation of effects and intervals of ethanol vs. all controls. exp(coef) corresponds to the risk ratio, that is, the possibility of reduced survival compared to ethanol. Concordance = 0.813 (se = 0.029); likelihood ratio test = 78.98 on 3 df, *p* ≤ 2 × 10^−16^; Wald test = 60.2 on 3 df, *p =* 5 × 10^−13^; score (logrank) test = 102.8 on 3 df, *p ≤* 2 × 10^−16^.

**Table 3:** Proportional risks model dose 8 mg/mL vs. the other doses evaluated.

Treatment	Coef	Se(coef)	Exp(coef)	Lower .95	Zpper .95	Z	Pr(>|z|)	Sig.
Doses 15 mg/mL ^a^	1.72352	0.06194	5.6042	4.9635	6.328	27.824	<2 × 10^−16^	0.001
Doses 10 mg/mL ^ab^	1.65814	0.06197	5.2495	4.6491	5.928	26.756	<2 × 10^−16^	0.001
Doses 14 mg/mL ^b^	1.44334	0.06278	4.2348	3.7445	4.789	22.991	<2 × 10^−16^	0.001
Doses 13 mg/mL ^bc^	1.24593	0.06389	3.4762	3.067	3.94	19.502	<2 × 10^−16^	0.001
Doses 9 mg/mL ^c^	1.04782	0.06442	2.8514	2.5132	3.235	16.266	<2 × 10^−16^	0.001

Contrast of the estimation of effects and intervals of the dose of 8 mg/mL vs. the other doses evaluated. exp(coef) corresponds to the risk ratio, that is, the possibility of reducing survival. Treatments with different letters show significant differences *p* < 0.05. Concordance = 0.713 (se = 0.004); likelihood ratio test = 2366 on 16 df, *p* ≤ 2 × 10^−16^; Wald test = 2187 on 16 df, *p* ≤ 2 × 10^−16^; score (logrank) test = 2385 at 16 df, *p* ≤ 2 × 10^−16^.

**Table 4:** Cox proportional hazard model applied to synthetized coumarins.

Coumarin	Coef	Se(coef)	Exp(coef)	Lower .95	Upper .95	z	Pr(>|z|)	Sig.
C4 ^a^	1.74347	0.07073	5.71712	4.9771	6.567	24.651	<2 × 10^−16^	0.001
C8 ^b^	1.15293	0.07195	3.16747	2.7508	3.647	16.024	<2 × 10^−16^	0.001
C3 ^bc^	0.95753	0.07388	2.60526	2.2541	3.011	12.961	<2 × 10^−16^	0.001
C7 ^c^	0.80074	0.07436	2.22718	1.9251	2.577	10.769	<2 × 10^−16^	0.001
C9 ^de^	0.38424	0.07855	1.4685	1.259	1.713	4.892	9.99 × 10^−7^	0.001
C5 ^e^	0.20725	0.08073	1.23029	1.0502	1.441	2.567	0.0103	0.05
C2 ^ef^	0.15984	0.08052	1.17332	1.002	1.374	1.985	0.0471	0.05
C6 ^f^	−0.15629	0.08645	0.85532	0.722	1.013	−1.808	0.0706	0.1

Contrast of the estimation of effects and intervals of coumarin 1 vs. the other coumarins evaluated. exp(coef) corresponds to the risk ratio, that is, the possibility of reducing survival. Treatments with different letters show significant differences *p* < 0.05. Concordance= 0.713 (se = 0.004); likelihood ratio test = 2366 on 16 df, *p* ≤ 2 × 10^−16^; Wald test = 2187 on 16 df, *p* ≤ 2 × 10^−16^; score (logrank) test = 2385 at 16 df, *p* ≤ 2 × 10^−16^.

**Table 5:** Results of IC_50_, binding energy and residues involved in the best pose of coumarin on triosephosphate isomerase.

Compound	R3	R4	R5	R7	IC_50_ (mg/mL)	Binding Energy (kcal/mol)	Residues Involved
**1**	H	CH_3_	H	OH	14.14 ± 0.5	−6.56	Ser-158, Val-160, Arg-205
**2**	H	CH_3_	OH	OH	7.89 ± 0.6	−6.72	Asp-105, Lys-103, Glu-77, Glu-104, Asp-65, Phe-102, Lys-112
**3**	CH_3_	CH_3_	H	OH	10.52 ± 0.4	−6.90	Lys-112, Asn-65, Glu-77, Asp-105
**4**	CH_3_	CH_3_	OH	CH_3_	8.78 ± 0.3	−6.62	Lys-112, Asn-65, Glu-77, Phe-102
**5**	H	CH_2_Cl	H	OH	13.98 ± 1.1	−6.77	Gln-64, Cys-43, Glu-77, Ile-78
**6**	H	CH_2_Cl	OH	CH_3_	15.25 ± 0.7	−6.75	Lys-112, Glu-104, Glu-77, Phe-102
**7**	H	Phenyl	H	H	9.31 ± 0.5	−5.91	Glu-77, Ile-78
**8**	H	CH_3_	H	3-methoxyprop-1-ene	7.24 ± 0.3	−5.57	Ala-45, Gln-64
**9**	H	CH_3_	H	3-phenylprop-2-en-1-yl	10.94 ± 0.9	−7.99	Asn-65, Cys-66, Tyr-67, Arg-98

IC_50_ values were estimated from the dose–response model exhibiting the best fit according to the Akaike Information Criterion (AIC). The corresponding values were subsequently determined by numerical optimization using the Solver tool in Microsoft Excel.

## Data Availability

The data presented in this study are available within the article and Supplementary Materials. Additional data are available from the corresponding author upon reasonable request.

## Supplementary Materials

The following supporting information can be downloaded at: https://www.mdpi.com/article/10.3390/molecules31173100/s1, Table S1: Experimental and predicted acaricidal activity values for coumarin derivatives and proposed structures. Table S2: Numerical values of the molecular descriptor used in the QSAR model.

## Abbreviations

The following abbreviations are used in this manuscript:

Abbreviation	Definition
TIM	Triosephosphate isomerase
QSAR	Quantitative structure–activity relationship
IC_50_	Half maximal inhibitory concentration
HR	Hazard ratio
CONICET	Consejo Nacional de Investigaciones Científicas y Técnicas
APC	Article Processing Charge
