# Supplementary material for: Green Synthesis, Molecular Docking, and QSAR Study of Coumarin Derivatives as Acaricidal Agents Against Rhipicephalus (Boophilus) microplus
Source: Molecules. 2026 Sep 4;31(17):3100. doi: 10.3390/molecules31173100 (PMC13567135; doi:10.3390/molecules31173100)
Supplement: Supplementary file 1 [file molecules-31-03100-s001.zip › molecules-4368888-supplementary.pdf]

Green Synthesis, Molecular Docking, and QSAR Study of Coumarin Derivatives as Acaricidal Agents against *Rhipicephalus (Boophilus) microplus*

Carlos E. Rodriguez[1], Diego M. Ruiz[2], Alejandra Rodrigueza[1,3], Pablo R. Duchowicz[4], Gustavo P. Romanelli[2,5], José J. Martínez[6] and Laura Juliana Triana Triana[7]

Table S1. Experimental and QSAR predicted (Eq. 1) $\log IC_{50}$ acaricidal activity for coumarine derivatives. Test set members are denoted with ^. Warning leverage=0.857.

ID	Compound name	Exp.	Eq. 1	Residual	Leverage
1		1.150	1.034	0.117	0.144
2		0.897	0.890	0.007	0.135
3		1.022	1.069	-0.047	0.146
4 ^		0.943	0.981	-0.038	0.140
5 ^		1.146	1.220	-0.074	0.156
6		1.183	1.179	0.004	0.154
7		0.969	0.998	-0.029	0.142
8		0.860	0.875	-0.015	0.134
9		1.039	1.075	-0.036	0.147
10		-	0.952	-	0.139
11		-	0.894	-	0.135
12		-	1.006	-	0.142
13		-	1.244	-	0.158
14		-	1.093	-	0.148
15		-	1.038	-	0.144

16		-	1.188	-	0.154
17		-	1.112	-	0.149
18		-	1.193	-	0.155
19		-	1.100	-	0.148
20		-	1.182	-	0.154
21		-	0.763	-	0.126
22		-	0.850	-	0.132
23		-	0.944	-	0.138
24		-	1.059	-	0.146
25		-	1.067	-	0.146
26		-	1.084	-	0.147
27		-	0.835	-	0.131
28		-	0.821	-	0.130
29		-	0.721	-	0.124
30		-	0.729	-	0.124
31		-	0.690	-	0.122
32		-	0.793	-	0.128
33		-	1.163	-	0.153
34		-	1.163	-	0.153
35		-	1.135	-	0.151
36		-	1.061	-	0.146
37		-	1.182	-	0.154
38		-	1.249	-	0.159

**39**		-	1.134	-	0.151
**40**		-	1.043	-	0.144
**41**		-	1.193	-	0.155
**42**		-	0.748	-	0.126
**43**		-	0.801	-	0.129
**44**		-	0.788	-	0.128
**45**		-	0.873	-	0.133
**46**		-	0.875	-	0.134
**47**		-	0.836	-	0.131
**48**		-	0.763	-	0.126
**49**		-	0.721	-	0.124
**50**		-	0.690	-	0.122

Table S2. The best selected molecular descriptor predicting the acaricidal activity.

ID	d_1
1	0.867186
2	0.838523
3	0.874115
4	0.856714
5	0.904352
6	0.896281
7	0.860033
8	0.835445

9	0.875413
10	0.850885
11	0.839147
12	0.861713
13	0.909244
14	0.878943
15	0.868073
16	0.897918
17	0.882850
18	0.899041
19	0.880409
20	0.896757
21	0.812972
22	0.830355
23	0.849188
24	0.872141
25	0.873774
26	0.877134
27	0.827481
28	0.824637
29	0.804660
30	0.806229
31	0.798374
32	0.819121
33	0.893089
34	0.893089
35	0.887404
36	0.872667
37	0.896757

38	0.910215
39	0.887121
40	0.869008
41	0.899041
42	0.810057
43	0.820679
44	0.817964
45	0.835122
46	0.835445
47	0.827662
48	0.812972
49	0.804660
50	0.798374
